# Decoction of Chinese Herbal Medicine Fuzheng Kang-Ai Induces Lung Cancer Cell Apoptosis via STAT3/Bcl-2/Caspase-3 Pathway

**DOI:** 10.1155/2018/8567905

**Published:** 2018-06-25

**Authors:** Sumei Wang, Shunqin Long, Shujing Xiao, Wanyin Wu, Swei Sunny Hann

**Affiliations:** ^1^Department of Oncology, Guangdong Provincial Hospital of Chinese Medicine, The Second Clinical Medical College, Guangzhou University of Chinese Medicine, Guangzhou, Guangdong 510120, China; ^2^The Postdoctoral Research Station, Guangzhou University of Chinese Medicine, Guangzhou, Guangdong 510120, China; ^3^Guangdong Provincial Key Laboratory of Clinical Research on Traditional Chinese Medicine Syndrome, Guangzhou, Guangdong, China; ^4^Laboratory of Tumor Biology, Guangdong Provincial Hospital of Chinese Medicine, The Second Clinical Medical Collage, Guangzhou University of Chinese Medicine, Guangzhou, Guangdong 510120, China

## Abstract

Decoction of Chinese herbal medicine (CHM) Fuzheng Kang-Ai (FZKA for short) has been applied as adjuvant treatment strategy in advanced lung cancer patients for decades. We previously showed that FZKA decoction inhibited proliferation of non-small cell lung cancer (NSCLC) cells through activation of AMP-activated protein kinase alpha (AMPK*α*) signaling pathway, followed by inducing insulin-like growth factor (IGF) binding protein 1 (IGFBP1) and forkhead homeobox type O3a (FOXO3a) proteins, and enhanced the inhibition effect of gefitinib in lung cancer cell growth via inactivating PI3-K/Akt-mediated suppressing of cell surface-associated mucin-1 (MUC1) expression. In this study, we investigated the molecular mechanism by which FZKA decoction affected cell apoptosis in lung cancer cells. Our results show that FZKA induced apoptosis in lung cancer cells. Mechanistically, FZKA activated the caspase-3, PARP, and caspase-9 activities. Both antiapoptotic and proapoptotic proteins from Bcl-2 family were deregulated by FZKA exposure in lung cancer cells. In addition, FZKA reduced protein expressions of signal transducer and activator of transcription 3 (STAT3) and Jun activation domain-binding protein 1 (Jab1), while it concomitantly increased p21 protein. Moreover, the inhibitor of caspase-3 resisted the effect of FZKA on induction of apoptosis. Finally, exogenous overexpression of STAT3 overcame FZKA-inhibited protein expressions of Bcl-2 and myeloid cell leukemia-1 (Mcl-1) as well as Bax and blocked FZKA-induced activities of caspase-3 and caspase-9. Our results show that FZKA decoction promotes lung cancer cell apoptosis through STAT3/Bcl-2/caspase-3 signaling pathways. This study unveils potential novel molecular mechanism by which FZKA controls growth of human lung cancer cells.

## 1. Introduction

Lung cancer is a leading cause of cancer-related death in the world [1]. In China, lung cancer is the most frequently diagnosed cancer in males (22.14%) and is the leading cause of cancer death in both males (27.21%) and females (21.91%) [2]. Despite major advances in combination treatments, the prognosis of non-small cell lung cancer (NSCLC) is still dismal and the 5-year survival rate with all stages and subtypes combined remains as low as 11% [3]. The Chinese herbal medicine (CHM) provides promising strategy for patients with late stage of lung cancer. In the last decades, many traditional Chinese herbs (TCH) with antitumor properties have drawn more attention due to their substantial efficacy, less drug resistance, and minimal toxicities, resulting in increase in quality of life and patient survival [4, 5]. For example, Yangfei Kongliu Formula (YKF) combined with cisplatin significantly inhibited the growth and metastasis of lung cancer cells via TGF-*β*1 pathway [6]. Homeopathic Psorinum-6x triggered apoptosis in lung cancer A549 cells thorough both upregulation and downregulation of relevant signal proteins, including p53, caspase-3, Bax, and Bcl-2 [7]. However, the molecular mechanism underlying the therapeutic potential has not been well elucidated.

Fuzheng Kang-Ai (FZKA) decoction, a 12-herb traditional formula, was firstly prescribed by Dr. Wanyin Wu and has been used to treat NSCLC patients in Guangdong Provincial Hospital of Chinese Medicine for decades, which showed a positive impact on patients. Previous study showed that FZKA combined with gefitinib resulted in longer progression-free survival (PFS) with less toxicity than gefitinib alone [8]. Meanwhile, FZKA could enhance the disease control rate (DCR) as well as median survival time (MST) in NSCLC patients [9, 10]. Mechanistically, we also found that FZKA inhibited growth of NSCLC cells through AMP-activated protein kinase alpha- (AMPK*α*-) mediated induction and interplay of insulin-like growth factor (IGF) binding protein 1 (IGFBP1) and forkhead homeobox type O3a (FOXO3a), demonstrating definite therapeutic effect in lung cancer [11]. More recently, we have observed that FZKA decoction inhibits the growth of NSCLC cells through phosphatidylinositol-3-kinase (PI3-K)/protein kinase B (Akt)-mediated inhibition of Nuclear factor-*κ*B (NF-*κ*B) subunit p65, followed by reducing expression of cell surface-associated mucin-1 (MUC1). More importantly, the combination of FZKA decoction and gefitinib, an epidermal growth factor receptor tyrosine kinase inhibitor (EGFR-TKI), has a synergistic effect on NSCLC cells. The* in vitro* and* in vivo *studies have provided a potential novel mechanism by which the FZKA decoction enhances the growth inhibition of gefitinib in gefitinib-resistant NSCLC cells [12]. Herein, we provided more evidences to demonstrate the proapoptotic effects of FZKA in lung cancer cells.

Caspase proteins, a group of cysteine proteases, have been well defined as key drivers in the process of cell apoptosis. Study reported that diallyl trisulfide (DATS), a constituent of processed garlic, could induce apoptosis of prostate cancer cell through enhancing the activities of caspase-3 and caspase-9 proteins, which was inhibited in the presence of caspase-3- or caspase-9-specific inhibitors, Z-VAD-FMK and Z-LEHD-FMK [13]. Another group also showed that artesunate, a derivative of artemisinin extracted from* Artemisia annua*, induced apoptosis through caspase-dependent mitochondrial pathway [14], suggesting the critical role of the caspase pathway in this process. The Bcl-2 protein family are large apoptosis regulatory proteins that modulate the mitochondrial pathway [15]. The increase of Bcl-2 protein family member Bax leads to the release of cytochrome C and other proapoptotic molecules from intermembranous space to cytosol, resulting in activation of downstream caspases [16]. Among those, caspase-3 is a frequently activated death protease, which cleaves poly(ADP-ribose) polymerase (PARP), a DNA repair enzyme [17]. Transcription factor signal transducer and activator of transcription 3 (STAT3) has been shown to be closely associated with not only growth but also apoptosis in cancer. STAT3 regulated cell survival by inducing Bcl-2 and Bcl-XL to repress apoptosis; thus degradation and inhibition of STAT3 increased apoptosis in several cancer cells [18, 19]. Nevertheless, the detailed mechanism underlying those complicated regulation axes, especially how those molecules modulate cell apoptosis and control lung cancer cell growth, still remains to be elucidated. In the current study, we performed experiments to understand the molecular mechanism by which FZKA induced apoptosis in lung cancer cells.

## 2. Materials and Methods

### 2.1. Fuzheng Kang-Ai Decoction (FZKA Decoction)

Decoction of FZKA, a Chinese herbal medicine (CHM) prescribed at the clinic, obtained from Guangdong Kangmei pharmaceutical Company, Ltd. (Guangdong, China), has been used to treat NSCLC in Guangdong Provincial Hospital of Chinese Medicine for more than 10 years.  Table 1 listed the components of this decoction and the FZKA decoction has been reported previously [10]. The batch to batch consistency studies (the chromatograms in FZKA decoction) using high-performance liquid chromatography (HPLC) analysis and chemical profiling of main constituents in FZKA decoction using ultra-high pressure liquid chromatography coupled with LTQ Orbitrap mass spectrometry have been reported previously [11]. All of the components from this prescription were soaked for 30 min before decoction. The concentration liquid was finally spray-dried into particles by Guangdong One Pharmaceutical Co., Ltd. For* in vitro* experiments, the granules were dissolved in RPMI-1640 medium to a final concentration of 20 mg/mL and centrifuged at 14,000 rpm for 10 min; the supernatant was then filtered with 0.22 *μ*m filter before use and the PH value of the cultured cells with media was adjusted to 7.2–7.4 after FZKA addition.

### 2.2. Chemicals and Cell Culture

Monoclonal antibodies specific of caspase-3, PAPR, caspase-9, Bcl-2, myeloid cell leukemia-1 (Mcl-1), Bax, Jun activation domain-binding protein 1 (Jab1), total STAT3 and the phosphor forms, and p21 were purchased from Cell Signaling Technology, Inc. (Beverly, MA, USA). Caspase-3 and caspase-9 activity assay kits were ordered from Abcam (Cambridge, MA, USA). Lipofectamine 3000 reagent was purchased from Life Technologies (AB & Invitrogen) (Carlsbad, CA, USA). STAT3 overexpression vector was obtained from OriGene Technologies, Inc. (Rockville, MD, USA). Dimethyl sulfoxide (DMSO) was purchased from Sigma-Aldrich Co. (St. Louis, MO, USA). NSCLC cells A549 were obtained from the Cell Line Bank at the Laboratory Animal Center of Sun Yat-sen University (Guangzhou, China) and PC9 and H1650 were obtained from the Chinese Academy of Sciences Cell Bank of Type Culture Collection (Shanghai, China). All cells were grown at 37°C in a humidified 5% CO_2_ and 95% air and cultured in RPMI-1640 medium (Life Technologies, Carlsbad, CA, USA) containing 10% FBS (Gibco, USA) and 0.5% penicillin-streptomycin sulfate (Invitrogen Life Technologies, Carlsbad, CA, USA). Cells were counted using the automated cell counter star (Inno-Alliance Biotech Inc., Denver, CO, USA).

### 2.3. High-Performance Liquid Chromatography (HPLC) Analysis

The initial batch to batch consistency study was performed using HPLC. Briefly, the samples solutions were put into the HPLC system (250 × 4.6 mm, 5 *μ*m, ACE, Scotland). The mobile phase consisted of deionized water with 0.1% formic acid (A) and acetonitrile with 0.1% formic acid (B). The gradient elution program was as follows: 5% B at 0–5 min, 5–20% B at 5–10 min, 20–40% B at 10–15 min, 40–95% B at 15–40 min, and 95–100% B at 40–45 min. The flow rate was 1.0 mL/min, and the detection wavelength was set at 280 nm. The injection volume was 10 *μ*L and the column temperature was maintained at 30°C.

### 2.4. Flow Cytometry Analysis

Cell apoptosis was analyzed by Annexin V-FITC/PI Apoptosis Detection Kit according to the manufacturer's protocol (Sigma-Aldrich Co., St. Louis, MO). Briefly, cells (H1650, A549, and PC9) were seeded in 6-well plates. After 24 h of culture, cells were treated with increased doses of FZKA and then incubated at 37°C for 24 h. Afterwards, cells were collected, centrifuged for 5 min at 1500 rpm, and resuspended in 1x binding buffer. Finally, 5 *μ*L Annexin V-FITC and 5 *μ*L PI were added to the cells at room temperature for 15 min. The cells were then analyzed using flow cytometer (Beckman FC 500, Beckman Coulter, Inc., CA, USA).

### 2.5. Caspase-3/9 Activity Assay

Caspase-3/9 activities were measured using caspase-3/9 activity assay kit according to manufacturer's protocol (Cell Signaling Technology, Inc., Beverly, MA, USA). In brief, lung cancer cells treated with FZKA were harvested and lysed in lysis buffer (4°C for 30 min). Equal amounts of formulations (100 *μ*L) were loaded onto a 96-well microplate and incubated at 37°C for 120 min with reaction buffer and the liberated DEVD-p-NA substrate. Afterwards, the caspase-3/9 activity was measured using the microplate reader at a wavelength of 405 nm and the caspase activities were expressed as percentage of enzyme activity compared to that in the control (untreated cells).

### 2.6. Western Blot Analysis

Briefly, the cells were harvested, washed, and lysed with 1x RAPI buffer. Protein concentration was determined by the Thermo BCA protein assay kit. Equal amounts of protein from cell lysates were solubilized in 5x SDS sample buffer and separated on 8–10% SDS polyacrylamide gels and transferred onto polyvinylidene fluoride membranes. Membranes were blocked with 5% non-fat milk in TBST and incubated with primary antibodies against caspase-3, PAPR, caspase-9, Bcl-2, Mcl-1, Bax, total STAT3 and the phosphor form, Jab1, and p21 proteins at 4°C overnight. Afterwards, the membranes were washed and incubated with a secondary antibody against rabbit IgG for 1 h, followed by washing and transferring into ECL solution (Millipore, Darmstadt, Germany), and scanned under the Bio-Rad ChemiDoc XRS+ chemiluminescence imaging system (Bio-Rad, Hercules, CA, USA). The results were measured by ImageJ software.

### 2.7. Transient Transfection Assays

The cells were seeded in 6-well plates and reached to 50–60% confluence. The control, siRNA, and STAT3 overexpression vectors were obtained from OriGene Technologies, Inc. (Rockville, MD, USA). For each well, 2–4 *μ*g of STAT3 plasmid DNA constructs or 10–60 nM STAT3 siRNA was transfected into the cells using Lipofectamine 3000 reagent (Life Technologies, Carlsbad, CA, USA) for 24 h based on the instruction from the provider, followed by treatment with FZKA for an additional 24 h for all other experiments.

### 2.8. Statistical Analysis

Statistical analysis was performed using the SPSS statistical software. Statistical evaluation for data analysis used Student's *t*-test when there were only two groups (two-sided) and differences between groups were assessed by one-way ANOVA. All data are reported as means ± SD. Differences between groups were considered statistically significant when *P* ≤ 0.05.

## 3. Results

### 3.1. Different Drinks of FZKA Decoction Have Similar Patterns

**Figure 1:**
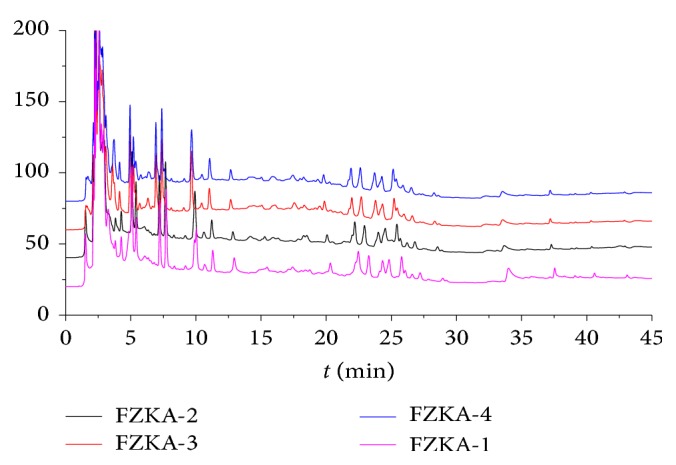
HPLC chromatograms in different drinks of FZKA decoction have similar patterns. The water extraction of the compound prescriptions of the different groups of the FZKA decoction was qualitatively analyzed by HPLC method as described in Section 2. Conditions: column: C_18_ column (250 × 4.6 mm, 5 *μ*m); rate: 1.0 mL/min; column temperature: 30°C; injection volume: 10 *μ*L. Four batches of FZKA decoction water extracts including a mixture of three different batches (FZKA 1–4) are presented.

We performed HPLC (high-performance liquid chromatography) to identify the main components in the different drinks of FZKA decoction. Our results showed that HPLC chromatograms in four drinks of FZKA decoction have similar patterns, revealing that the FZKA decoction has a good stability (Figure 1).

### 3.2. FZKA Induces Lung Cancer Cell Apoptosis

**Figure 2:**
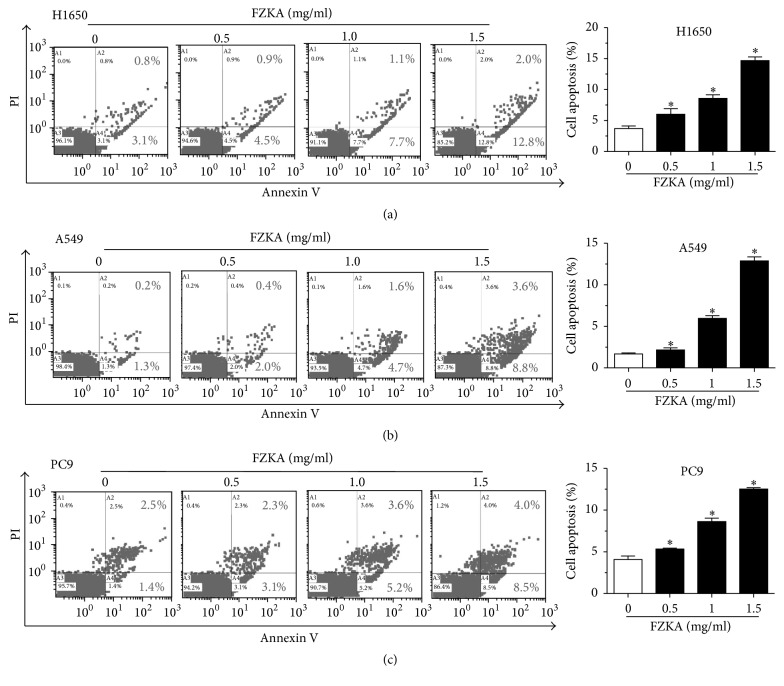
*FZKA induces lung cancer cell apoptosis*. (a–c) Lung cancer cells (H1650, A549, and PC9) were treated with increased concentrations of FZKA for 24 h. Afterwards, cells were harvested for analysis of apoptosis using the Annexin V-FITC/PI Apoptosis Detection Kit as detailed in Materials and Methods. The A3 quadrant (annexin V−/PI−), A4 quadrant (annexin V+/PI−), and A2 quadrant (annexin V+/PI+) of the histograms indicated the percentage of normal cells, early apoptosis, and late apoptosis, respectively. Values in bar graphs were given as the mean ± SD from three independent experiments performed in triplicate. *∗* indicates significant difference as compared to the untreated control group (^*∗*^*P* < 0.05).

Our previous studies have shown that FZKA decoction inhibited lung cancer cell growth through multiple mechanisms [11, 12]. We then explored the potential mechanism by which FZKA affected apoptosis in NSCLC cells. Flow cytometry of cell apoptosis was performed when treating NSCLC cells (H1650, A549, and PC9) with different doses of FZKA for 24 h. Our results showed that FZKA significantly induced the apoptosis in NSCLC cells (more than 50% when treated with 1.5 mg/mL FZKA) in a dose-dependent manner as shown in the right (A2 and A4) quadrants of the histograms, which were counted as apoptotic cells detected by the Annexin V-FITC/PI Apoptosis Detection Kit. After 24 h of treatment, the FZKA-induced apoptotic rate was greater than that in the nontreated control cells (Figures 2(a)–2(c)).

### 3.3. FZKA-Stimulated Activation of Caspase-3/9 Contributes to the Induction of Apoptosis in Human Lung Cancer Cells

**Figure 3:**
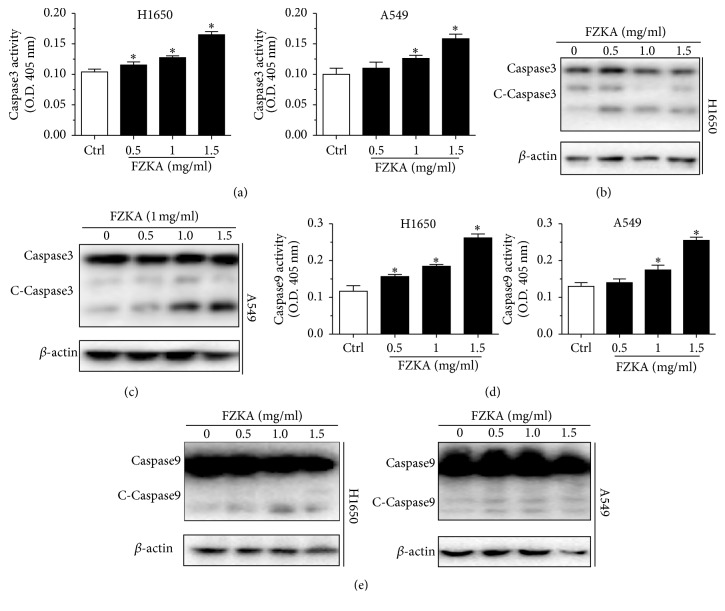
*FZKA-stimulated activation of caspase-3/9 contributes to the induction of apoptosis in human lung cancer cells*. (a) Caspase-3 activity was detected using caspase-3 activity assay kit in H1650 and A549 cells treated with FZKA as indicated doses for 24 h. Data represent means ± SD of three independent experiments. *∗* indicates significant difference as compared to the untreated control group (^*∗*^*P* < 0.05). (b-c) The protein expression levels of caspase-3 and c-caspase-3 were detected by Western blot. *β*-Actin was used as an internal control. The results were measured by ImageJ software. (d) Caspase-9 activity was detected using caspase-9 activity assay in H1650 and A549 cells treated with indicated doses of FZKA for 24 h. Data represent means ± SD of three independent experiments. *∗* indicates significant difference as compared to the untreated control group (^*∗*^*P* < 0.05). (e) The protein expression levels of caspase-9 and c-caspase-9 were detected by Western blot. *β*-Actin was used as an internal control.

To identify the relevant targets of FZKA-induced apoptosis, we started to examine the effect of FZKA on the activation of caspases. We observed that FZKA increased the activation of caspase-3 in a dose-dependent fashion in H1650 and A549 cells (Figure 3(a)). In line with this, the Western blot experiments also showed activation of caspase-3 by inducing cleaved-caspase-3 (c-caspase-3) protein in H1650 and A549 cells (Figures 3(b) and 3(c)). Furthermore, we then tested whether the induction of apoptosis by FZKA is associated with extrinsic or intrinsic apoptosis pathway. We found that caspase-9, a key component of intrinsic apoptosis pathway, was significantly activated by the FZKA treatment as shown by cleaved-caspase-9 (c-caspase-9) protein levels in H1650 and A549 cells, suggesting that the apoptotic effect of FZKA is mediated at least in part by the extrinsic apoptosis pathway (Figures 3(d) and 3(e)).

### 3.4. Bcl-2 Family Is Involved in the FZKA-Induced Lung Cancer Cell Apoptosis

**Figure 4:**
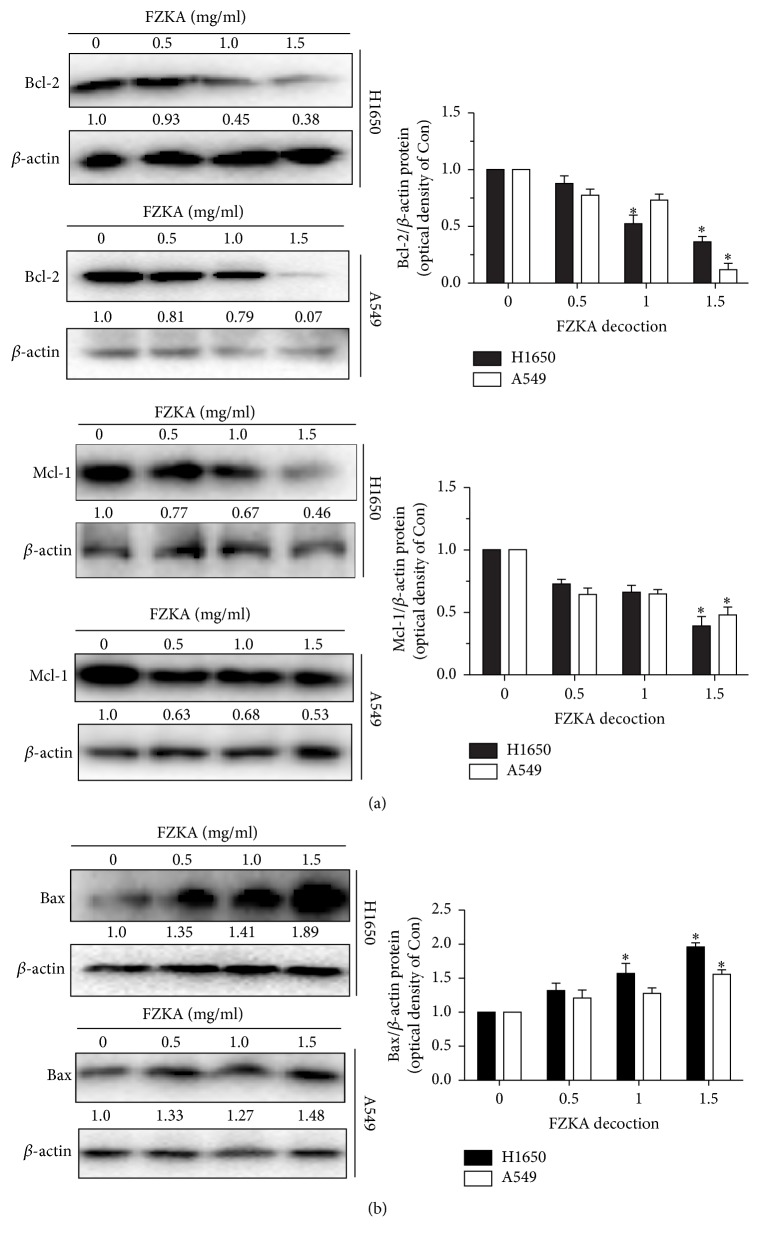
*Bcl-2 family is involved in the FZKA-induced lung cancer cell apoptosis*. (a-b) The protein expression levels of Bcl-2, Mcl-1, and Bax were detected by Western blot. *β*-Actin was used as an internal control. The results were measured by ImageJ software. Values in bar graphs were given as the mean ± SD from three independent experiments. *∗* indicates significant difference as compared to the untreated control group (*P* < 0.05).

The apoptosis signaling can be initiated either at the cell surface through a death receptor-induced signaling pathway or within the cell via the release of proapoptotic molecules, such as Bcl-2 family proteins. Caspases are linked to Bcl-2 family which is the key regulator of apoptosis in cancer [20]. To further clarify the molecular mechanisms underlying FZKA-induced apoptosis in lung cancer cells, the expressions of Bcl-2 family members including Bcl-2, Mcl-1, and Bax in H1650 and A549 cells treated with FZKA were examined. The results demonstrated that the expression of Bcl-2 and Mcl-1 (two antiapoptotic proteins) was decreased, whereas the expression of Bax (proapoptotic protein) was increased in a dose-dependent manner (Figures 4(a) and 4(b).). The above results suggested that the apoptosis induced by FZKA was mediated through mitochondria-dependent pathway as well as changes in the protein expressions of Bcl-2 families.

### 3.5. STAT3/Jab1/p27 Pathways Play a Role in the Process of FZKA-Induced Lung Cancer Cell Apoptosis

**Figure 5:**
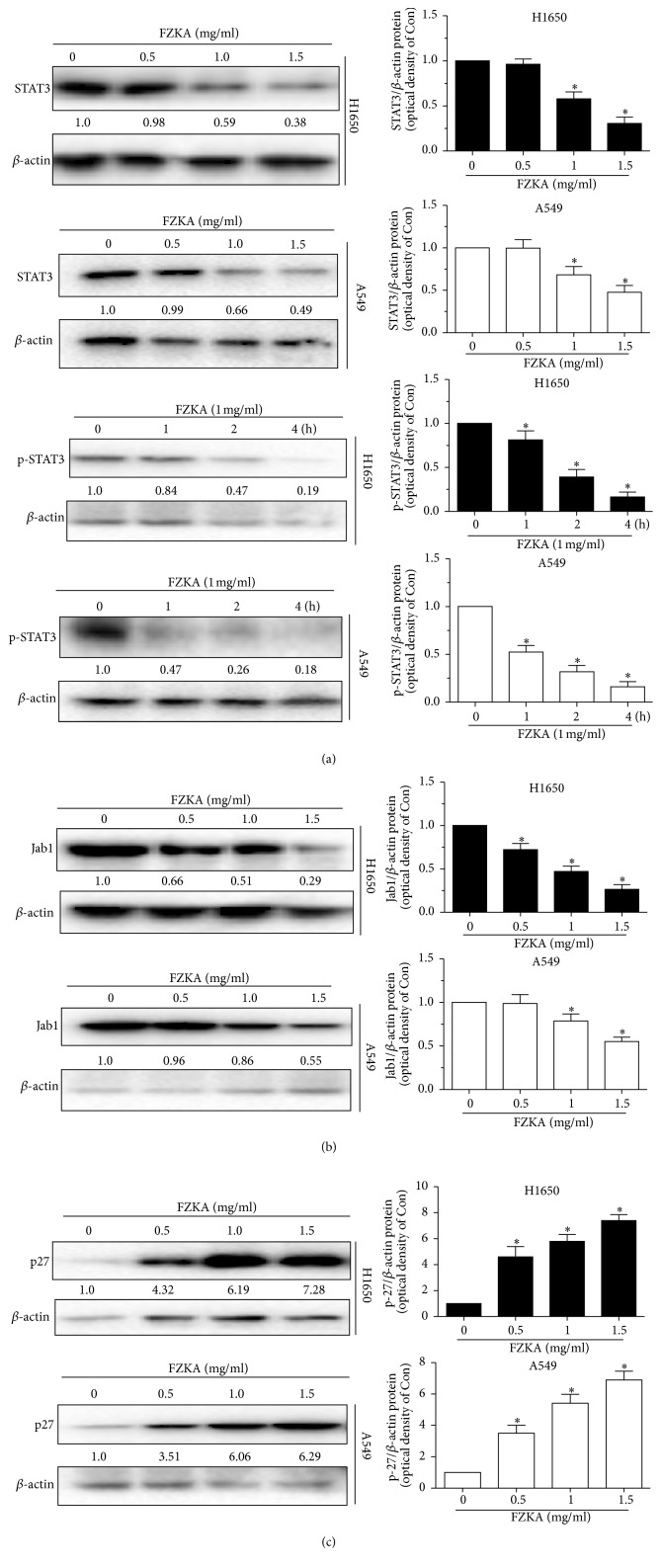
*STAT3/Jab1/p27 pathways play a role in the process of FZKA-induced lung cancer cell apoptosis*. (a–c) A549 and H1650 cells were treated with indicated doses of FZKA for up to 4 h and cell lysate was harvested, and the expressions of the phosphorylated or total protein of STAT3, Jab1, and p27 were measured by Western blot analysis using corresponding antibodies. *β*-Actin was used as an internal control. Data were measured by ImageJ software. Values in bar graphs were given as the mean ± SD from three independent experiments. *∗* indicates significant difference as compared to that in the untreated control group (^*∗*^*P* < 0.05).

STAT3 has been reported to modulate the expression of genes involved in antiapoptosis, such as Bcl-2 and Mcl-1. We then tested the expression of STAT3 by FZKA treatment. Our results showed that STAT3 expression was decreased by FZKA treatment in dose-dependent manner, and the phosphorylation of STAT3 was also inhibited by FZKA in a time-dependent manner (Figure 5(a)). Jab1/CSN5 (c-Jun activation domain-binding protein 1, Jab1 hereafter) was originally identified as a c-Jun coactivator and subsequently discovered to be one of downstream molecules of STAT3, which functions as an oncoprotein in cancers [21]. In the present study, Jab1 was found to be downregulated by FZKA treatment (Figure 5(b)). On the contrary, p27, a cell cycle inhibitor and one of the targets of Jab1 [22], was unregulated by FZKA in H1650 and A549 cells in a dose-dependent manner (Figure 5(c)). The above data indicated that the STAT3/Jab1/p27 pathways might be involved in the FZKA-induced apoptosis in lung cancer cells.

### 3.6. Activation of Caspase-3 Mediates FZKA-Induced Apoptosis

**Figure 6:**
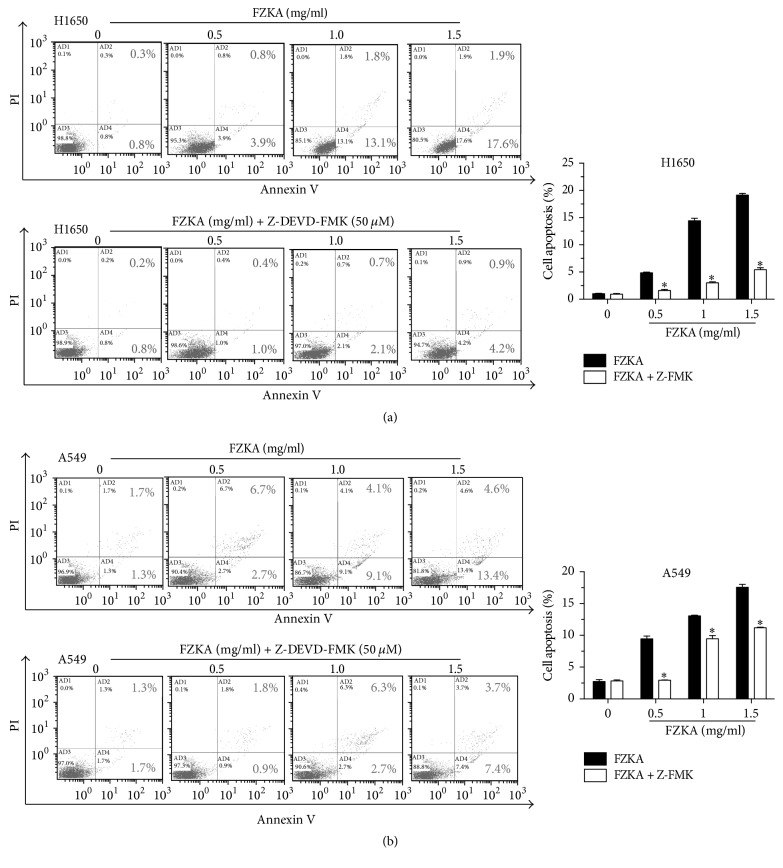
*Activation of caspase-3 mediates FZKA-induced apoptosis*. (a-b) H1650 and A549 cells were seeded into 6-well plates and treated with increased doses of FZKA or the pan-caspase inhibitor Z-VAD-FMK at 50 *μ*M for 1 h before exposing the cells to FZKA for an additional 24 h. Afterwards, cells were harvested for analysis of apoptosis using the Annexin V-FITC/PI Apoptosis Detection Kit as detailed in Materials and Methods. The AB3 quadrant (annexin V−/PI−), AB4 quadrant (annexin V+/PI−), and AB2 quadrant (annexin V+/PI+) of the histograms indicated the percentage of normal cells, early apoptosis, and late apoptosis, respectively. Values in bar graphs were given as the mean ± SD from three independent experiments performed in triplicate. *∗* indicates significant difference as compared to the untreated control group (*P* < 0.05).

To further interrogate whether caspases activation is required for the induction of apoptosis, we pretreated cells with the pan-caspase inhibitor Z-VAD-FMK at 50 *μ*M for 1 h before exposure of the cells to FZKA for an additional 24 h. Our results showed that the percentages of cell apoptosis of the group pretreated with Z-VAD-FMK as detected by the Annexin V-FITC/PI Apoptosis Detection Kit described in Materials and Methods were much lower as compared to those in the control group without Z-VAD-FMK treatment in H1650 and A549 cells (Figures 6(a) and 6(b)). This data indicated that FZKA-induced cell apoptosis was at least in part through the activation of caspase-3 pathway.

### 3.7. STAT3 Knock-Down Enhances the Effect of FZKA on the Activities of Caspase-3, Caspase-9, and Bcl-2 Families

**Figure 7:**
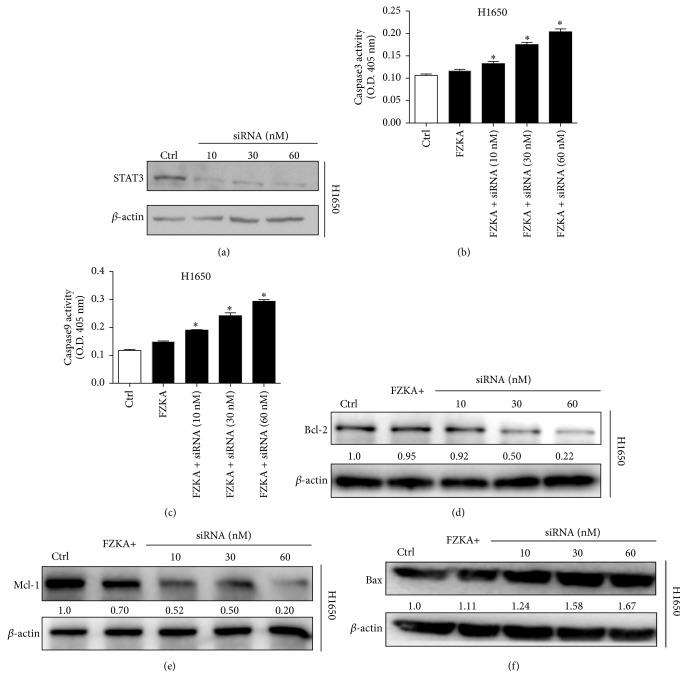
*STAT3 knock-down enhanced the effect of FZKA on the activities of caspase-3, caspase-9, and Bcl-2 families*. (a) STAT3 siRNA and control were transfected to H1650 cells and STAT3 expression was decreased in a dose-dependent manner. (b-c) H1650 cells were transfected with control or STAT3 siRNA for 24 h, followed by exposure of the cells to FZKA for an additional 24 h. Afterwards, caspase-3 and caspase-9 activities were detected using caspase-3/9 activity assay as described in Materials and Methods. Data represent means ± SD of three independent experiments. *∗* indicates significant difference as compared to the untreated control group (*P* < 0.05). (d–f) H1650 cells were transfected with control or STAT3 siRNA for 24 h, followed by exposure of the cells to FZKA for an additional 24 h. The protein expression levels of Bcl-2, Mcl-1, and Bax were detected by Western blot. *β*-Actin was used as an internal control.

To clarify the important role of STAT3 in the apoptosis process of FZKA on lung cancer cells, we decreased the expression of STAT3 by STAT3 siRNA transfection (Figure 7(a)). Our results showed that the combination of FZKA treatment with STAT3 knock-down increased the activities of both caspase-3 and caspase-9 in lung cancer cells, compared to FZKA alone (Figures 7(b) and 7(c)). Moreover, the expressions of Bcl-2 family including Bcl-2, Mcl-1, and Bax were also enhanced by STAT3 knock-down with the combination by FZKA, compared to FZKA alone (Figures 7(d)–7(f)). These results indicated the critical role of STAT3-mediation effect on FZKA-induced apoptosis in lung cancer cells.

### 3.8. Overexpression of STAT3 Overcomes the Effect of FZKA on the Activities of Caspase-3, Caspase-9, and Bcl-2 Families

**Figure 8:**
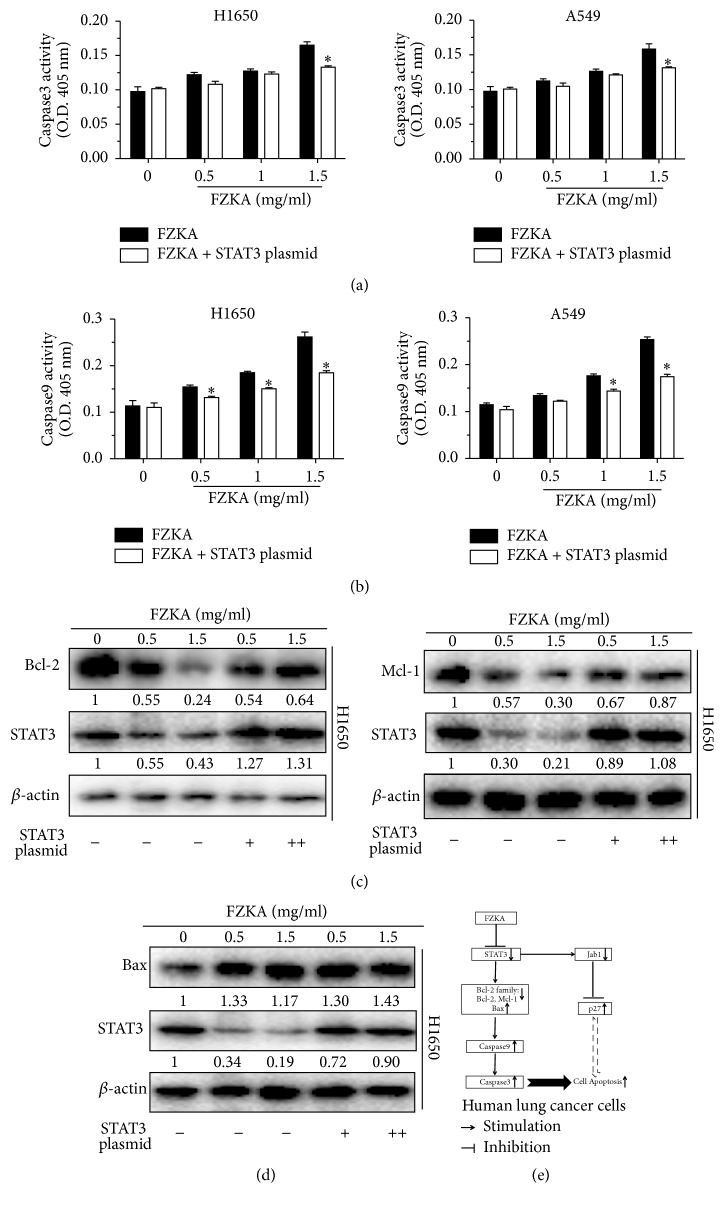
*Overexpression of STAT3 overcame the effect of FZKA on the activities of caspase-3, caspase-9, and Bcl-2 families*. (a-b) H1650 and A549 were transfected with control or STAT3 overexpression plasmids for 24 h, followed by exposure of the cells to FZKA for an additional 24 h. Afterwards, caspase-3 and caspase-9 activities were detected using caspase-3/9 activity assay as described in Materials and Methods. Data represent means ± SD of three independent experiments. *∗* indicates significant difference as compared to the untreated control group (*P* < 0.05). (c-d) H1650 and A549 cells were transfected with control or STAT3 overexpression plasmids for 24 h, followed by exposure of the cells to FZKA for an additional 24 h. The protein expression levels of Bcl-2, Mcl-1, and Bax were detected by Western blot. *β*-Actin was used as an internal control. (e) A schematic diagram shows that FZKA induces lung cancer cell apoptosis through inactivation of STAT3, followed by reducing antiapoptotic proteins Bcl-2 and Mcl-1, while inducing proapoptotic protein Bax. This activates caspase-3/9 and concomitantly increases p27 expression, thereby leading to cell apoptosis. Our findings uncover an additional mechanism underlying the induction of apoptosis by FZKA and suggest that the FZKA-induced lung cancer cell apoptosis is mainly through activation of intrinsic apoptosis pathway.

To further clarify the functional relevance of those potential targets involved in the regulation of cell apoptosis by FZKA treatment, we then decipher the role of STAT3 in this process. Our data showed that exogenous expression of STAT3 overcame the FZKA-induced activities of caspase-3 and caspase-9 in H1650 and A549 cells (Figures 8(a) and 8(b)). In line with these results, we also observed that the decreased expression of Bcl-2 and Mcl-2 as well as the increased Bax protein expression by FZKA was blocked in cells overexpressing STAT3 exogenously (Figures 8(a) and 8(d)). Collectively, these above results implied that STAT3 might be an upstream molecule of Bcl-2 family affected by FZKA in this process.

## 4. Discussion

In the present study, we proposed to uncover the role and mechanisms by which FZKA decoction affects apoptosis in lung cancer cells. We found that FZKA exerted the cytotoxic effects via the induction of apoptosis in lung cancer cells. At present, there are two widely accepted classical pathways of cell apoptosis: mitochondrial pathway and death receptor pathway [23, 24]. Mitochondria play a key role in the process of cell apoptosis as they can regulate and participate in the whole process of tumor cell apoptosis [25]. During cell apoptosis, the apoptotic pathway is reflected in the aspects of modification of mitochondrial transmembrane voltage and increase of caspase activity induced by the openness of mitochondrial outer membrane permeability (MOMP) [26]. Our results showed that FZKA induced the cleavage of caspase-9 and caspase-3 in lung cancer cells. When caspase-3 was inhibited by its inhibitor Z-VAD-FMK, cell apoptosis induced by FZKA could be blocked. This data strongly suggested that the FZKA-induced apoptosis was through a mitochondrial pathway in a caspase-3-dependent manner.

It has been reported that Bax can transfer to mitochondrial outer membrane along cytoplasm and thus enhance the permeability of mitochondrial membrane. Therefore, when the level of Bax increases and transmembrane voltage steps down, proteins located in mitochondria such as cytochrome can be released so that a series of reactions are brought about and cell apoptosis occurs in tumor cells [26–28]. Another study showed that bufalin, a cardiotonic steroid, reduced the expressions of antiapoptotic protein Bcl-2 and surviving and increased the proapoptotic protein Bax/Bcl-2 rate in several different cancer types [29]. Consistent with this, we demonstrated that FZKA induced expression of Bax, while it inhibited the expression of Bcl-2 and Mcl-1 from Bcl-2 family. The data presented herein suggested that Bcl-2 family mediated the FZKA-induced cell apoptosis in a mitochondrion-dependent manner.

Moreover, FZKA induced lung cancer cell apoptosis significantly with concomitant induction of p27, reduction of Jab1 protein expression, and phosphorylation of STAT3. Transcription factor STAT3 is often constitutively activated in various human cancers and controls the expression of multiple genes involved in tumor initiation, growth, progression, and apoptosis [30, 31]. Once activated, STAT3 undergoes phosphorylation-induced homodimerization, leading to nuclear translocation, DNA binding, and subsequent gene transcription. Regulation of STAT3 has been reported to modulate the expression of genes involved in cell apoptosis, such as Bcl-2 family [32]. Thus we reasoned that STAT3 regulated the expression of Bcl-2 family in the FZKA-treated lung cancer cells. Our results demonstrated that exogenously overexpressed STAT3 abolished FZKA-induced apoptosis, implying the role of STAT3 expression in this process. Those findings provided evidence and suggested that STAT3/Bcl-2/caspase-3 signaling pathways were involved in the FZKA-induced apoptosis in NSCLC cells. On the other hand, Jab1, acting as a modulator of intracellular signaling and affecting cellular proliferation and apoptosis and a target of STAT3 [33, 34], also decreased in FZKA-treated NSCLC cells. Meanwhile, in the current study, FZKA-treated NSCLC cells showed an increase in protein expression of p27, a cyclin-dependent kinase (Cdk) inhibitor, which results in cell-cycle arrest [35]. This data indicated that STAT3/Jab1/p27 pathways might contribute to the FZKA-induced lung cancer cell apoptosis. Collectively, these above findings uncovered a novel mechanism by which FZKA decoction acted as a tumor suppressive compound and inhibited lung cancer cell growth. More experimental approaches are still required to further elucidate the molecular mechanism underlying this proapoptotic process by FZKA.

## 5. Conclusions

In our study, we investigated the effects of FZKA on the cell apoptosis in human lung cancer cells. Our results show that FZKA induces apoptosis of H1650 and A549 cells via inhibition of STAT3, followed by increasing protein expression of Bax, concomitantly decreasing Bcl-2 and Mcl-1 protein levels, and significantly activating caspase-9 and caspase-3. The overall effects of FZKA were significantly abrogated in cells transfected with exogenously overexpressed STAT3. Taken together, these results uncover an additional mechanism by which FZKA controls human lung cancer cell growth.

## Figures and Tables

**Table 1:** Components of “Fuzheng Kang-Ai” (FZKA) decoction.

Chinese name	Common name	Weight (g)
Tai Zi Shen	Radix Pseudostellariae	30
Bai Zhu	Rhizoma Atractylodis Macrocephalae	15
Huangqi	Milkvetch Root	30
Baihuasheshecao	*Hedyotis diffusa*	30
Long Kui	*Solanum nigrum*	30
Shi Jian Chuan	Chinese Sage Herb	30
Shancigu	Indian Iphigenia Bulb	30
Yi Yi Ren	Coix seed	30
Bayuezha	*Akebia trifoliata* Koidz.	30
Shepaole	Snake bubble ilicifolius	30
Ezhu	*Curcuma zedoaria*	15
Gan Cao	Licorice	10
